# Comparative Assessment of Predictive Models for Older Adults’ Health Outcomes: Logistic Regression, LASSO, ML, LLM

**DOI:** 10.1093/geroni/igaf122.2244

**Published:** 2025-12-31

**Authors:** Feng Wei, Yuanhao Liu, Jiayu Zheng

**Affiliations:** Johns Hopkins University, Baltimore, Maryland, United States; Johns Hopkins University, Baltimore, Maryland, United States; Johns Hopkins University, Baltimore, Maryland, United States

## Abstract

Accurate prediction of health outcomes in older adults is crucial for tailoring interventions and informing public health strategies. Despite advancements in predictive methodologies, significant gaps remain in understanding how these models perform across various health indicators and demographic segments. This study evaluates four predictive methodologies—conventional logistic regression, regularization represented by LASSO, machine learning ensembles (decision trees, random forests, support vector machines), and AI LLM (DeepSeek and GPT)—focusing on two critical health outcomes: depression and mortality. Using nationally representative data from the Health and Retirement Study (HRS) and the China Health and Retirement Longitudinal Study (CHARLS), we evaluate these models’ performance and fairness comparatively: (1) evaluate model performance through traditional metrics such as accuracy, precision, recall, F1-score, and ROC-AUC; (2) explore whether performance discrepancies are consistent across these health outcomes; (3) investigate fairness by examining predictive performance disparities among elderly subpopulations defined by geographic location, gender, and socioeconomic status, using fairness metrics like statistical parity, equalized odds, and predictive parity. Through this multidimensional comparison, our study provides a comprehensive evaluation of the progression from traditional to AI-based predictive models, contributes to understanding the current capabilities and limitations in predicting elderly health outcomes, and identifies the trade-offs between model complexity, predictive accuracy, and fairness. These potential outcomes are expected to inform further research, and provide practical guidance for policymakers, educators, and healthcare practitioners in deploying equitable and effective predictive strategies for elderly health outcomes.

